# Dynamic succession of substrate-associated bacterial composition and function during *Ganoderma lucidum* growth

**DOI:** 10.7717/peerj.4975

**Published:** 2018-06-13

**Authors:** Bo Zhang, Lijuan Yan, Qiang Li, Jie Zou, Hao Tan, Wei Tan, Weihong Peng, Xiaolin Li, Xiaoping Zhang

**Affiliations:** 1 Department of Microbiology, College of Resources, Sichuan Agricultural University, Chengdu, China; 2 Soil and Fertilizer Institute, Sichuan Academy of Agricultural Sciences, Chengdu, China; 3 Chair for Aquatic Geomicrobiology, Institute of Biodiversity, Friedrich Schiller University Jena, Jena, Germany; 4 Biotechnology and Nuclear Technology Research Institute, Sichuan Academy of Agricultural Sciences, Chengdu, China; 5 College of Life Sciences, Sichuan University, Chengdu, China

**Keywords:** *Ganoderma lucidum*, Dynamic change, NGS, *G. lucidum*–bacteria-substrate, Functional pathways, Bacterial composition

## Abstract

**Background:**

*Ganoderma lucidum*, a valuable medicinal fungus, is widely distributed in China. It grows alongside with a complex microbial ecosystem in the substrate. As sequencing technology advances, it is possible to reveal the composition and functions of substrate-associated bacterial communities.

**Methods:**

We analyzed the bacterial community dynamics in the substrate during the four typical growth stages of *G. lucidum* using next-generation sequencing.

**Results:**

The physicochemical properties of the substrate (e.g. acidity, moisture, total nitrogen, total phosphorus and total potassium) changed between different growth stages. A total of 598,771 sequences from 12 samples were obtained and assigned to 22 bacterial phyla. *Proteobacteria* and *Firmicutes* were the dominant phyla. Bacterial community composition and diversity significantly differed between the elongation stage and the other three growth stages. LEfSe analysis revealed a large number of bacterial taxa (e.g. *Bacteroidetes*, *Acidobacteria* and *Nitrospirae*) with significantly higher abundance at the elongation stage. Functional pathway prediction uncovered significant abundance changes of a number of bacterial functional pathways between the elongation stage and other growth stages. At the elongation stage, the abundance of the environmental information processing pathway (mainly membrane transport) decreased, whereas that of the metabolism-related pathways increased.

**Discussion:**

The changes in bacterial community composition, diversity and predicted functions were most likely related to the changes in the moisture and nutrient conditions in the substrate with the growth of *G. lucidum*, particularly at the elongation stage. Our findings shed light on the *G. lucidum*-bacteria-substrate relationships, which should facilitate the industrial cultivation of *G. lucidum*.

## Introduction

*Ganoderma lucidum* belongs to the phylum *Basidiomycota*, and its growth mainly depends on lignin as a carbon source (Mizuno et al., 1995). The fruiting bodies and spores of *G. lucidum* are highly appreciated as health products in China for their richness in polysaccharides and triterpenoids, which are demonstrated to strengthen the immune system and inhibit tumor formation (Wang et al., 2002; Sakamoto et al., 2016). Because of its high medicinal value, the planting area of *G. lucidum* is expanding. *G. lucidum* has become the main economic pillar in some places due to the advantages of its cost-effective production management, e.g., requiring a small investment, having a short life cycle and yielding benefits fast (Boh et al., 2007). Like other edible fungi, the growth of *G. lucidum* depends on many environmental factors (e.g., temperature, enzyme activity and microbial community), which likely induce changes in the content of nutrients such as polysaccharides and microelements in its fruiting bodies (Stajic et al., 2002; Tanaka et al., 2016). Li et al. (2014) demonstrated that the nutrient content in different tissues of *G. lucidum* changed with its growth and that it attributed to the extracellular enzyme activities. Significant growth-related differences were present in crude polysaccharides and triterpenes in the fruiting bodies of most of the *G. lucidum* strains that were tested (Fu et al., 2008). A greater number of trace elements and heavy metals were found in the mycelia than in the fruiting bodies or spores of *G. lucidum* (Xing et al., 2001). Studies have characterized the subtle changes of various substances in the body of *G. lucidum* during its growth; however, changes in microbial community in the surrounding cultivating environment or in the substrate have seldom been studied.

As previously reported, bacteria in the surrounding soil or the culture media were likely to change into endophytic bacteria, and play an important role in the growth of edible fungi (Gagne et al., 1987; Elvira & Van, 2000). The endophytic bacteria assist their host with nitrogen fixation, growth promotion and disease resistance (Mano & Morisaki, 2008; Zhang, 2010; Wang, Chen & Peng, 2016). Qiu et al. (2011) revealed that a variety of microorganisms existed in the mushroom substrate and significantly affected the host development. In particular, tiny changes in microbial communities in the culture substrates may impact the growth and development of edible fungi (Cho et al., 2003). Ma, Chen & Chen (2016) characterized the significant allelopathic effects of the dominant microbes (mainly molds and bacteria) on the growth of *G. lucidum* in a continuous cropping soil using culture-based methods and demonstrated that bacteria such as *Clostridium*, *Alkaligenes* and *Bacillus* had stronger allelopathic effects on *G. lucidum*. In addition, pollution rates were associated with changes in microbial communities in the industrial production of *Pleurotus eryngii* (Lin et al., 2010).

The DNA-based community-fingerprinting methods, such as DGGE and T-RFLP (Székely et al., 2009), are cost-effective ways to explore the changes in microbial community structure in the environment. Nevertheless, these methods lack a clear description of the microbial taxonomy and tend to underestimate microbial diversity with a relatively low resolution. Currently, next-generation sequencing technology has overcome these issues and is widely utilized to explore the distribution of microorganisms in diverse ecological conditions, including freshwater lakes, marine water, agriculture soil, forest soil, thermal vents and even in some valuable herbs (Patel & Jain, 2015; Gigliotti et al., 2015; Xia et al., 2016; Meier, 2016; Ávila et al., 2016; Su et al., 2017).

Previous studies on *G. lucidum* were mainly related to the cultivation technology and the active components in fruiting bodies. There have been few reports on the dynamic changes in microbial communities in the substrate at different growth stages of *G. lucidum*. In this study, next-generation sequencing of the V3–V4 region of bacterial 16S rRNA gene was used to determine the composition and diversity of bacterial communities and to predict the potential functions of the dominant microorganisms in the substrate during the four growth stages of *G. lucidum* (hyphal stage, budding stage, elongation stage and mature stage).

## Materials and Methods

### Cultivation of *Ganoderma lucidum*

The *Ganoderma lucidum* cultivar Chuan Yuanzhi No. 1, provided by the Soil and Fertilizer Institute at the Sichuan Academy of Agricultural Sciences, has been deposited in the China General Microbiological Culture Collection Center (CGMCC) with the strain number of CGMCC 13174 on October 21st, 2016. The substrate was composed of cottonseed hull (90%), wheat bran (5%), corn flour (4%) and gypsum (1%) (Fig. S1), all of which were fresh, dry and unspoiled. The substrate was put into polypropylene cultivation bags (size: 17 cm × 33 cm × 0.005 cm). One side of the polypropylene bag was covered with breathable paper for air entry during the cultivation of *G. lucidum*. The cultivation bags were then autoclaved at 121 °C for 2 h. The purpose of autoclaving was to create a relatively aseptic environment for mycelium germination. The existence of microbes in the substrate would likely acidify the substrate, affecting the germination and growth of *G. lucidum*. Therefore, it was a good cultivation practice to sterilize the substrate before inoculation of the fungus *G. lucidum*. After sterilization, the bags were cooled to room temperature and placed in a laminar flow cabinet for inoculation of *G. lucidum* (Fig. S2). A small piece of colonised grain (approximately 80 g) that was filled with mycelium of *G. lucidum* with great vitality was inoculated into each cultivation bag. After inoculation, the cultivation bags were placed in a greenhouse in the cultivation site at Zhaojia, Jintang, China (N 30°48′16.45″, E 104°35′48.79″). The space of the cultivation site had been previously ventilated, cleaned and simply disinfected with lime before the experiment was carried out. The cultivation bags were arranged in parallel in two rows and two layers with seven to eight bags in each on the ground in the greenhouse and the edge of each group was reinforced with wooden piles (Fig. S3).

The sampling of *G. lucidum* was done at the four growth stages: hyphal stage, budding stage, elongation stage and mature stage. After inoculation, the mycelia of *G. lucidum* began to germinate. The first sampling was done at the hyphal stage (approximately 35 days after the inoculation) when the mycelia of *G. lucidum* spread and subsequently filled the whole culture medium. The mycelia twisted together. The second sampling was done at the budding stage (approximately 46 days after the inoculation) when the primordia formed and started to differentiate. The third sampling was done at the elongation stage (approximately 56 days after inoculation) when the promordia grew longer and the stipe was formed. Cap differentiation began after elongation. The last sampling was done at the mature stage (approximately 66 days after the inoculation) when the spores appeared on the pileus surface and gradually covered the yellow edges. Disposable disinfected gloves, sterilized tweezers and knives were used for sampling. At each growth stage, three cultivation bags were brought to the lab and the substrate materials taken from different parts of each cultivation bag were pooled together and homogenized (Fig. S4). Finally, a total of twelve samples were collected in the four growth stages. The fresh samples were stored at −20 °C in 2 mL Eppendorf tubes prior to DNA extraction.

### Chemical analysis of substrate materials

Substrate materials at different growing stages of *G. lucidum* were collected and the chemical properties were determined including pH value, moisture, total nitrogen, total phosphorus and total potassium. The samples were first digested with sulfuric acid hydrogen peroxide. Then the treatment solution of each sample was analyzed with the conventional method according to Thomas, Sheard & Moyer (1967).

### DNA extraction, PCR amplification and MiSeq sequencing

No less than 500 mg of substrate materials per sample were collected for DNA extraction. Three biological replicates of samples taken at each growth stage were treated independently to ensure the methodological reproducibility. The E.Z.N.A.^®^ Soil DNA kit (OMEGA Bio-Tek, Norcross, GA, USA) was used to isolate DNA from the substrate following the manufacturer’s protocol. DNA concentration was measured using a UV spectrophotometer (Bio Photometer; Eppendorf, Hamburg, Germany). The quality and size of the extracted DNA was checked by 0.8% agarose gel electrophoresis.

The PCR amplification was performed by Shanghai Personal Biotechnology Co., Ltd (Shanghai, China). Both the primer information and the PCR protocol have been described in detail in Srinivasan et al. (2012). PCR amplification was performed using the bacterial 16S rRNA gene-specific primers 338F (5′-ACTCCTACGGGAGGCAGCA-3′) and 806R (5′-GGACTACHVGGGTWTCTAAT-3′) with the following conditions: 98 °C for 2 min (initial denaturation), 25 cycles of 98 °C for 15 s (denaturation), 55 °C for 30 s (annealing) and 72 °C for 30 s (extension), and 72 °C for 5 min (final extension) (Langenheder & Székely, 2011). The PCR products were purified with Agencourt AMPure Beads (Beckman Coulter, Indianapolis, IN) and quantified using a Quant-iT Pico Green dsDNA Assay Kit with a microplate reader (FLx800; Bio-Tek, Norcross, GA, USA) and were mixed based on the concentration of each sample. Amplicon sequencing was performed on Illumina’s MiSeq platform (Personalbio, Shanghai, China). The samples were barcoded before pooling. The barcodes and adapters were trimmed with FASTX Toolkit. All raw data were submitted to the Sequence Read Archive (SRA) database with the accession numbers SRR5801759–SRR5801768 and SRR5801783–SRR5801784.

### Sequence and statistical analysis

Reads containing ambiguous ‘N’ or with length <120 nt or >140 nt were discarded. High-quality sequences with 97% or greater similarity were clustered into OTUs using UCLUST (Edgar, 2010), a sequence alignment tool, using QIIME pipeline version 1.7.0 (Caporaso et al., 2010). All non-bacterial sequences were removed after classification. The most abundant sequence of each OTU was selected as the representative sequence of this OTU. The relative abundances of the OTUs were calculated. The OTUs with relative abundance lower than 0.001% of the total sequences across all samples were removed (Bokulich & Mills, 2013). The multivariate statistical analyses were done using the OTU relative abundance data in R environment (R Core Team, 2016). An unconstrained ordination (non-metric multidimensional scaling NMDS) was used to visualize the broad pattern of the distribution of bacterial communities. PERMANOVA was used to test the significance of the difference in bacterial communities between different growth stages of *G. lucidum* based on 999 permutations. Both NMDS and PERMANOVA analysis were performed based on weighted UniFrac distance using the R vegan package (McArdle & Anderson, 2001; Oksanen et al., 2008). The pairwise PERMANOVA was used to test the difference in the bacterial community composition between two growth stages at each time, when the growth stage was found significant to affect the bacterial community composition in the overall term PERMANOVA test. The numbers of shared OTUs were presented in a Venn diagram using the R VennDiagram package (Chen & Boutros, 2011). Bacterial alpha diversity indices including observed OTUs, Chao1, ACE, Shannon and Simpson were rarefied and calculated based on the smallest library size of the samples. LEfSe analysis (Segata et al., 2011) was used to reveal the bacterial taxa that showed differential abundance between different growth stages of *G. lucidum* at all taxonomic levels. PICRUSt software (Langille et al., 2013) was used to predict the metabolic functions of bacterial communities based on the microbial metabolic function categories in the KEGG database. All significant differences were concluded at *P* < 0.05.

## Results

### Chemical analysis of the substrate materials

The physicochemical properties of the substrate materials (e.g., acidity, moisture content, total nitrogen content, total phosphorus content and total potassium content) changed between the four growth stages of *G. lucidum* (Table 1). The substrate was acidic throughout the growth of *G. lucidum*. Substrate pH was lowest at the hyphal stage (4.34). After reaching a peak at the budding stage (5.29), the substrate pH decreased at the later growth stages. Substrate moisture content declined along with growth of *G. lucidum*. Particularly, the moisture content dropped sharply at the mature stage. We observed a lower total nitrogen content in the substrate at the budding and the elongation stages than at the hyphal and mature growth stages of *G. lucidum*. Total phosphorus and potassium in the substrate displayed the identical fluctuation pattern with the growth of *G. lucidum*. Both of them showed the highest content at the budding stage and the lowest content at the elongation stage.

**Table 1:** Chemical properties of substrate. Mean ± standard deviation. Statistical analysis was carried out by ANOVA using SPSS 19.0 software. The Post Hoc tests were done with LSD method. Different lower-case letters showed significant difference (*P* < 0.05) in the substrate chemical properties between the different growth stages of *G. lucidum*.

Growth stage	pH	Moisture (%)	Total nitrogen (g/kg)	Total phosphorus (g/kg)	Total potassium (g/kg)
Hyphal stage	4.34 ± 0.11 *c*	64.60 ± 1.08 *a*	13.53 ± 0.30 *a*	2.74 ± 0.13 *b*	13.31 ± 0.34 *b*
Budding stage	5.29 ± 0.15 *a*	58.01 ± 1.22 *b*	10.75 ± 0.29 *b*	3.36 ± 0.08 *a*	15.45 ± 0.13 *a*
Elongation stage	5.05 ± 0.01 *b*	54.48 ± 2.02 *c*	11.25 ± 0.59 *b*	1.64 ± 0.07 *d*	8.80 ± 0.19 *d*
Mature stage	4.89 ± 0.06 *b*	38.56 ± 1.54 *d*	12.60 ± 0.04 *a*	2.43 ± 0.03 *c*	11.47 ± 0.68 *c*

### Taxonomy-based analysis of bacterial community

**Figure 1:**
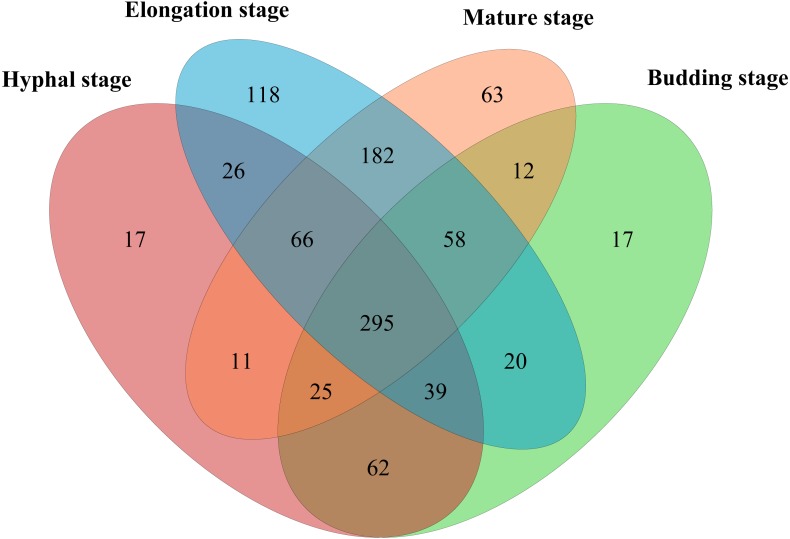
Venn diagram showing the number of shared OTUs between different growth stages of *G. lucidum*.

**Figure 2:**
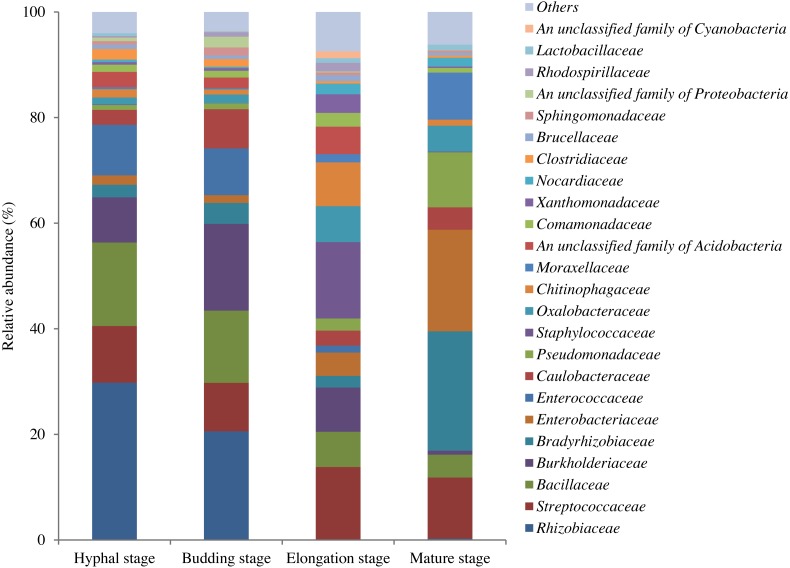
OTU average relative abundances of the major bacterial families in the substrate of *G. lucidum* during all growth stages. Others: the families with relative abundances lower than 1% at each growth stage of *G. lucidum*.

In total, 598,771 sequences from the 12 samples were clustered into 1200 OTUs at 97% similarity. A total of 15.7% of OTUs were unclassified at the phylum level. The high quality reads ranged from 25,996 to 69,618 OTUs between samples (Table S1). A total of 295 shared bacterial OTUs were found between the four growth stages of *G. lucidum* in the substrate (Fig. 1). A total of 22 phyla were detected in the substrate at the four growth stages of *G. lucidum*. As shown in Table 2, the most abundant phylum was *Proteobacteria*, which accounted for 41.47%–72.86% (average 57.23%) of all the bacterial sequences, followed by *Firmicutes* (22.50%–40.33%, average 34.12%). Together, these two phyla represented 80.12%–95.93% of the bacterial species. The less dominant phyla (average abundance >1%) included *Bacteroidetes* (3.30%), *Acidobacteria* (2.75%) and *Actinobacteria* (2.03%). A total of 195 genera were identified in the samples. Most of the identified genera belonged to the phyla of *Proteobacteria*, *Firmicutes*, *Bacteroidetes* and *Actinobacteria* (Table S2). Of the 24 major bacterial families (relative abundance >1%) showed in Fig. 2, 20 belonged to *Proteobacteria* and *Firmicutes*. Among these major families, two families of *Firmicutes* (e.g., *Streptococcaceae* and *Bacillaceae*) showed high relative abundance across all the growth stages. The relative abundance of proteobacterial families such as *Rhizobiaceae*, *Bradyrhizobiaceae* and *Enterobacteriaceae* changed greatly between the different growth stages.

**Table 2:** The average relative abundance of different bacterial phyla in the substrate during the four growth stages of *G. lucidum*. Others are all unclassified phyla.

Phylum	Abu. (%) at hyphal stage	Abu. (%) at budding stage	Abu. (%) at elongation stage	Abu. (%) at mature stage
[Thermi]	0.01	0.01	0.02	0.01
Acidobacteria	3.08	2.11	5.64	0.04
Actinobacteria	1.11	0.87	3.37	2.58
Bacteroidetes	1.65	1.00	8.66	1.48
Chloroflexi	0.00	0.00	0.03	0.01
Cyanobacteria	0.09	0.02	1.54	0.03
Elusimicrobia	0.01	0.00	0.02	0.01
Firmicutes	40.37	34.96	40.06	20.70
Fusobacteria	0.04	0.03	0.07	0.04
Gemmatimonadetes	0.01	0.01	0.04	0.00
Nitrospirae	0.00	0.00	0.03	0.00
Planctomycetes	0.01	0.00	0.03	0.00
Proteobacteria	53.6	60.99	40.43	74.93
Tenericutes	0.00	0.00	0.00	0.02
Verrucomicrobia	0.00	0.00	0.01	0.01
Others	0.01	0.00	0.06	0.14

### Bacterial alpha diversity

Bacterial alpha diversity indices significantly differed between the four growth stages of *G. lucidum* (Table 3). The richness indices (e.g., observed OTUs, Chao1 and ACE) were significantly higher at the late growth stages (e.g., elongation and mature stages) than at the early growth stages (e.g., hyphal and budding stages). The Shannon diversity was significantly higher at the elongation stage than at the hyphal stage. There was no difference in the Simpson index between the different growth stages of *G. lucidum.*

**Table 3:** Bacterial alpha diversity indices. Mean ± standard deviation. Statistical analysis was carried out by ANOVA using SPSS 19.0 software. The Post Hoc tests were done with LSD method. The index of the observed OTUs, was used to evaluate the observed OTU richness, whereas the Chao1 and ACE were used to estimate the total (observed and unobserved) OTU richness of the bacterial community. The indices of Shannon and Simpson were used to access the richness and evenness of bacterial community, respectively. Different lower-case letters showed significant difference (*P* < 0.05) in the diversity indices between the different growth stages of *G. lucidum*.

Sample	Observed OTUs	Chao1	ACE	Simpson	Shannon
Hyphal stage	363 ± 21 *b*	197.00 ± 21.66 *b*	260.22 ± 40.72 *b*	0.85 ± 0.10 *a*	4.02 ± 0.62 *b*
Budding stage	357 ± 13 *b*	189.00 ± 24.58 *b*	270.17 ± 35.29 *b*	0.88 ± 0.00 *a*	4.08 ± 0.09 *ab*
Elongation stage	505 ± 50 *a*	372.00 ± 70.55 *a*	479.66 ± 99.77 *a*	0.90 ± 0.06 *a*	4.77 ± 0.33 *a*
Mature stage	491 ± 33 *a*	336.67 ± 53.46 *a*	422.42 ± 56.42 *a*	0.87 ± 0.03 *a*	4.21 ± 0.13 *ab*

### Bacterial beta diversity

**Figure 3:**
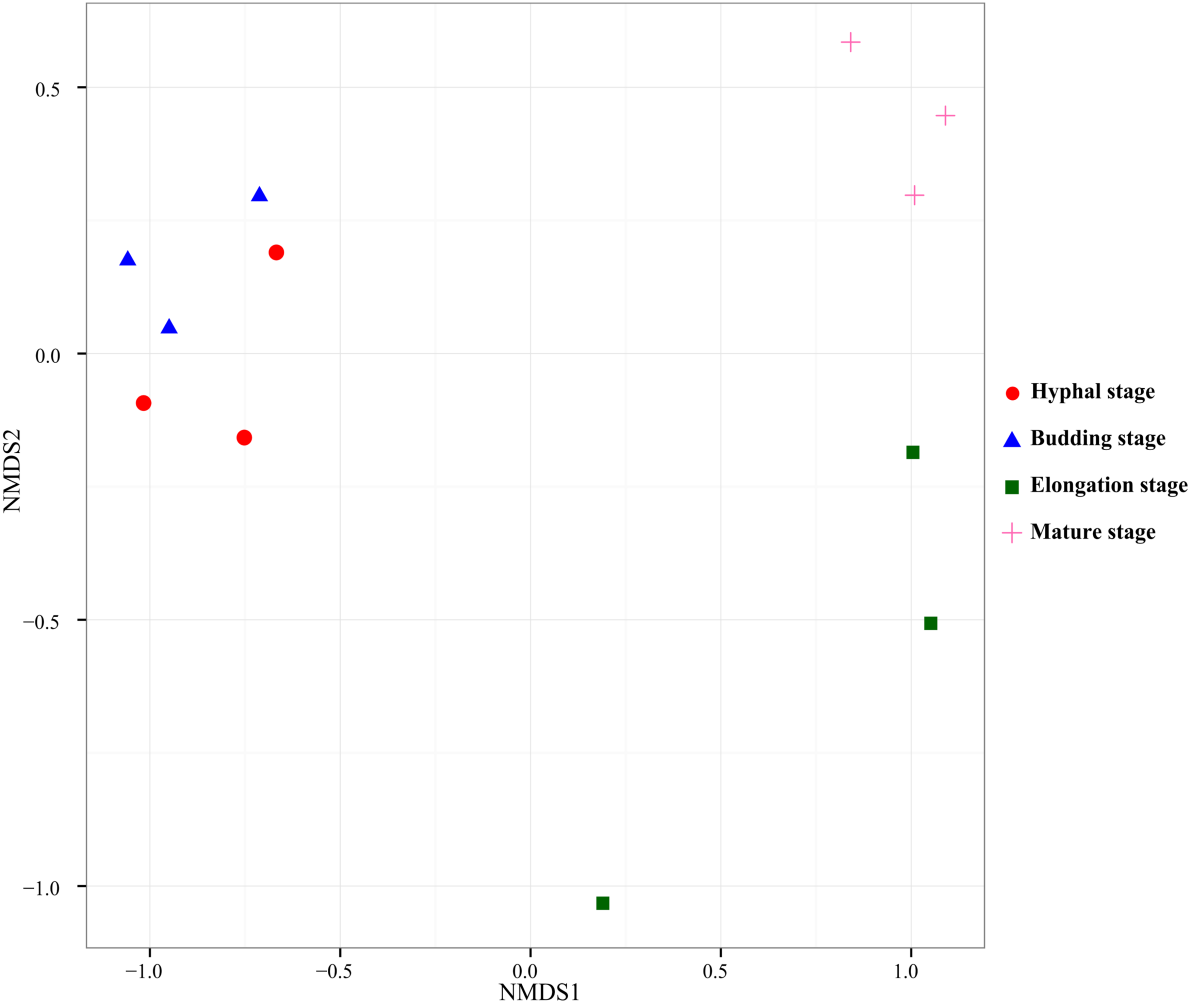
Nonmetric Multidimensional Scaling ordination of bacterial communities based on weighted UniFrac distance.

PERMANOVA test was used to test the effect of the growth stage of *G. lucidum* on the bacterial community composition in the substrate. According to the pairwise PERMANOVA test (Table 4), bacterial community composition significantly differed between the elongation stage and the other three growth stages. NMDS, an unconstrained ordination, was used to visualize the patterns of bacterial community distribution. The separation of the bacterial community samples at the elongation stage from the other three stages were clearly displayed in the NMDS ordination (Fig. 3). The bacterial community samples taken at the elongation stage were separated from the hyphal and budding stages on the first axis, whereas they were separated from the mature stage on the second axis in the NMDS ordination plot.

**Table 4:** PEMANOVA analysis of bacterial community between different growth stages based on weighted UniFrac distance. Significance of the differences in bacterial communities between the different growth stages were tested using 999 permutations.

		Significance
Hyphal stage	Budding stage	0.5014
	Elongation stage	0.0014
	Mature stage	0.1000
Budding stage	Elongation stage	0.0014
	Mature stage	0.1000
Elongation stage	Mature stage	0.0014
Hyphal stage, Budding stage, Elongation stage, Mature stage		0.0014

### Biomarker discovery

**Figure 4:**
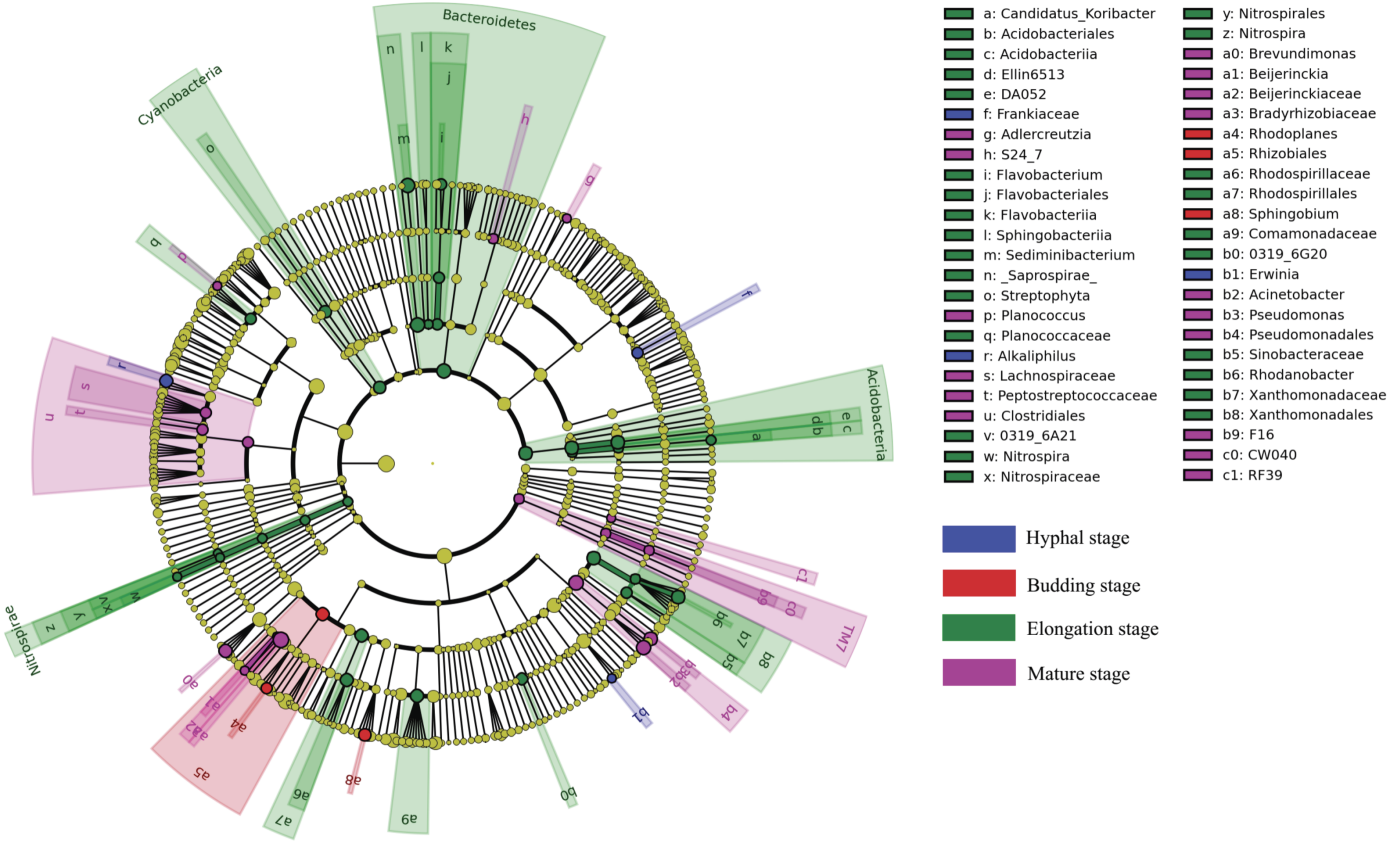
A cladogram showing the differentially abundant bacterial taxa at each of the four growing stages of *G*. *lucidum* based on LEfSe analysis (*P* < 0.05, LDA score > 2).

LEfSe analysis was used to reveal the bacterial taxa that showed differential abundances between the four different growth stages of *G. lucidum* at all taxonomic levels (Fig. 4). LEfSe analysis revealed a significantly higher abundance of the order *Rhizobiales* (e.g., genus *Rhodoplanes*) and the genus *Sphingobium* at the budding stage than at the other three growth stages of *G. lucidum*. The family *Frankiaceae* and the genera of *Alkaliphilus* and *Erwinia* were significantly enriched in the substrate at the hyphal stage, with regard to other growth stages. A large number of bacterial taxa exhibited a significantly higher abundance at the elongation stage. Those taxa included the phyla of *Bacteroidetes* (e.g., genera *Flavobacterium* and *Sediminibacterium*), *Acidobacteria* (e.g., genus *Candidatus Koribacter*), *Nitrospirae* (e.g., genus *Nitrospira*), *Cyanobacteria* (e.g., order *Streptophyta*) and other taxa such as the orders of *Xanthomonadales* and *Rhodospirillales* and the family of *Comamonadaceae*. The phylum of TM7 and the orders of *Clostridiales* (e.g., families *Peptostreptococcaceae* and *Lachnospiraceae*) and *Pseudomonadales* (e.g., genera *Acinetobacter* and *Pseudomonas*) were significantly more abundant at the mature stage than at other growth stages.

### Functional prediction of substrate bacteria community

A total of 39 KEGG pathways were identified in the study (Fig. S5). Of these KEGG pathways, 48.93% were related to metabolism, 17.24% to environmental information processing and 14.23% to genetic information processing. The ten most prevalent pathways were related to four function types: metabolism, environmental information processing, genetic information processing and unclassified. Membrane transport was the predominant KEGG pathway predicted by PICRUSt, accounting for an average of 15.93% during the growth of *G*. *lucidum*. Carbohydrate metabolism (on average 10.03%) and amino acid metabolism (on average 9.81%) were the second and the third most abundant pathways during the growth of *G*. *lucidum*.

The predicted pathways that had relative abundances over 1% at different growth stages of *G. lucidum* were presented in Table 5. A number of predicted bacterial functional pathways in the substrate were different between the elongation stage and the other three growth stages of *G*. *lucidum*. At the elongation stage, the relative abundance of membrane transport, and xenobiotics biodegradation and metabolism were decreased; other predicted pathways such as folding, sorting and degradation, replication and repair, translation, carbohydrate metabolism, energy metabolism, enzyme families, glycan biosynthesis and metabolism, and metabolism of cofactors and vitamins were enriched in the substrate bacterial community. Other pathways were not significantly different in abundance between different growth stages (e.g., cell motility, signal transduction, amino acid metabolism, and cellular processes and signaling).

**Table 5:** PICRUSt predicted KEGG pathways of bacterial community in the substrate (relative abundance  > 1% in at least one growth stage) at different growth stages of *G. lucidum*. Mean ± standard deviation. Statistical analysis was carried out by ANOVA using SPSS 19.0 software. The Post Hoc tests were done with LSD method. Different lower-case letters showed significant difference (*P* < 0.05) in the relative abundance of the pathways between the different growth stages of *G. lucidum*.

Function type	KEGG pathway	Relative abundance (%)			
		Hyphal stage	Budding stage	Elongation stage	Mature stage
Cellular processes	Cell motility	2.77 ± 0.05 *a*	2.97 ± 0.32 *a*	2.75 ± 0.32 *a*	2.95 ± 0.32 *a*
Environmental information processing	Membrane transport	17.40 ± 1.96 *a*	16.31 ± 1.80 *a*	11.36 ± 0.81 *b*	14.08 ± 0.72 *ab*
	Signal transduction	2.14 ± 0.05 *a*	2.20 ± 0.09 *a*	2.27 ± 0.05 *a*	2.35 ± 0.16 *a*
Genetic information processing	Folding, sorting and degradation	1.77 ± 0.10 *c*	1.79 ± 0.04 *bc*	2.18 ± 0.04 *a*	1.98 ± 0.11 *ab*
	Replication and repair	6.06 ± 0.27 *b*	5.99 ± 0.14 *b*	6.91 ± 0.15 *a*	6.06 ± 0.12 *b*
	Transcription	2.44 ± 0.10 *ab*	2.41 ± 0.02 *ab*	2.54 ± 0.09 *a*	2.26 ± 0.05 *b*
	Translation	3.46 ± 0.23 *b*	3.42 ± 0.12 *b*	4.11 ± 0.16 *a*	3.58 ± 0.07 *ab*
Metabolism	Amino acid metabolism	9.81 ± 0.16 *a*	10.11 ± 0.46 *a*	10.38 ± 0.12 *a*	10.07 ± 0.27 *a*
	Biosynthesis of other secondary metabolites	0.93 ± 0.02 *ab*	0.92 ± 0.04 *ab*	1.03 ± 0.11 *a*	0.80 ± 0.04 *b*
	Carbohydrate metabolism	10.07 ± 0.26 *b*	10.09 ± 0.11 *b*	10.68 ± 0.05 *a*	9.94 ± 0.28 *b*
	Energy metabolism	4.85 ± 0.07 *c*	4.80 ± 0.04 *c*	5.42 ± 0.05 *a*	5.05 ± 0.01 *b*
	Enzyme families	1.80 ± 0.09 *b*	1.77 ± 0.02 *b*	2.01 ± 0.05 *a*	1.67 ± 0.06 *b*
	Glycan biosynthesis and metabolism	1.76 ± 0.09 *b*	1.73 ± 0.04 *b*	2.18 ± 0.25 *a*	1.74 ± 0.15 *b*
	Lipid metabolism	3.75 ± 0.01 *a*	3.86 ± 0.19 *a*	3.89 ± 0.10 *a*	3.93 ± 0.10 *a*
	Metabolism of cofactors and vitamins	3.58 ± 0.01 *b*	3.58 ± 0.03 *b*	3.94 ± 0.14 *a*	3.74 ± 0.11 *ab*
	Metabolism of other amino acids	1.89 ± 0.04 *b*	2.02 ± 0.15 *ab*	1.94 ± 0.05 *ab*	2.13 ± 0.07 *a*
	Metabolism of terpenoids and polyketides	1.98 ± 0.03 *b*	2.06 ± 0.10 *b*	2.04 ± 0.04 *ab*	2.22 ± 0.07 *a*
	Nucleotide metabolism	2.97 ± 0.09 *a*	2.90 ± 0.14 *a*	3.16 ± 0.10 *a*	2.98 ± 0.05 *a*
	Xenobiotics biodegradation and metabolism	4.37 ± 0.20 *ab*	4.61 ± 0.28 *a*	3.73 ± 0.23 *b*	4.86 ± 0.42 *a*
Unclassified	Cellular processes and signaling	3.71 ± 0.13 *a*	3.73 ± 0.03 *a*	3.99 ± 0.09 *a*	3.89 ± 0.34 *a*
	Genetic information processing	1.98 ± 0.16 *b*	2.05 ± 0.08 *b*	2.45 ± 0.06 *a*	2.48 ± 0.18 *a*
	metabolism	2.72 ± 0.08 *a*	2.76 ± 0.04 *a*	2.72 ± 0.16 *a*	2.96 ± 0.12 *a*
	Poorly characterized	4.90 ± 0.12 *a*	4.91 ± 0.03 *a*	5.29 ± 0.07 *a*	5.13 ± 0.27 *a*

## Discussion

In this study, we observed significant changes in the physicochemical properties of the substrate materials during the growth of *G. lucidum*. The substrate materials together with the aeration and temperature used in our study provided an optimum condition for the cultivation of *G. lucidum.* For cultivation of edible and/or medicinal mushrooms, the substrate rich in essential bio-available nutrients with optimum pH, moisture, and air content is preferred (Chang & Wasser, 2018). Cottonseed hulls provide fine water holding capacity and nitrogen to ensure the water and nutrient supply for the cultivation of edible mushrooms such as *Pleurotus ostreatus* (Lu & Qu, 1984) and aerobic bacteria (Slifkin & Pouchet, 1975). The mixture of cottonseed hulls with other materials such as wheat bran, corn flour and gypsum was used for industrial production of *G. lucidum* in China. The growth of *G. lucidum* slightly increased substrate pH, especially at the budding stage. However, the substrate still remained acidic with pH values ranging between 4 and 5.5 at different growth stages. The moisture content continued declining with the vegetative growth of *G. lucidum*. The observed sharp decline of moisture content was likely associated with the development of the fruiting bodies at the mature stage, when the water demand was the largest. The lowered total nitrogen content in the substrate likely indicated the higher uptake of nitrogen of *G. lucidum* at the budding and elongation stages than at the hyphal and mature stages. This phenomenon might be related to the rapid mycelia growth and protein synthesis processes of *G. lucidum* at the later growth stages. Nitrogen content is one limiting factor that should be seriously taken into consideration during mushroom cultivation. Nitrogen supplementation improves the nutritional content in substrates (Lelley & Janssen, 1993), contributing to a higher yield of mushrooms (Yang, Chienyan & Chen, 2003). Dependent on the fungus physiology and the medium composition, nitrogen source was demonstrated to affect enzyme synthesis (Couto et al., 2004; Kapich et al., 2004). In addition, the C: N ratio can greatly impact the mycelia growth and fruiting body formation of *G. lucidum* (Hsieh & Yang, 2004)*.* It has been previously stated that more enzymes are involved in cell wall synthesis during fruiting body formation than at the mycelium and primordial stages. However, enzymes related to cell wall component degradation were higher expressed at the earlier stages of mushroom growth (Rahmad et al., 2014). Total phosphorus and potassium content in the substrate was lowest at the elongation stage, likely attributable to a high nutrient demand of *G. lucidum* at this stage. *G. lucidum* grows most prosperously at the elongation stage, a critical period for biomass and nutrient production. Minerals such as phosphorus and potassium were strongly correlated with the yield and biological efficiency of some edible fungi (e.g., *Ganoderma lucidum* and *Lentinula edodes*) (Ozcelik & Peksen, 2007; Peksen & Yakupoglu, 2009).

*Proteobacteria* and *Firmicutes* were the dominating phyla observed in the bacterial community in the substrate of *G. lucidum*, with variations in relative abundance between different growth stages of *G. lucidum*. *Proteobacteria* widely exist in high abundance in soils with diverse morphologies, physiologies and metabolisms and is considered to be advantageous in global carbon, nitrogen and sulfur cycling (Janssen, 2006; Kersters et al., 2006; Spain, Krumholz & Elshahed, 2009). A recent study suggested that the members of the phylum *Firmicutes* play important roles in the conversion of wheat straw into bio-utilizable sugars throughout the *Agaricus bisporus* mushroom cropping process (Mcgee et al., 2017). Durrer et al. (2016) characterized the role of biological connections in agricultural soils and revealed that competition among various bacteria may cause a decline in the abundance of some taxa. The relative abundance of *Firmicutes* decreased with time in our study, likely due to the reduced bioavailability of the wheat straw and the resource competition between other organisms in the substrate at the late growth stage of *G. lucidum*. Besides *Firmicutes*, other lignocellulose degrading bacteria belonging to *Actinobacteria*, *Proteobacteria* and *Bacteroidetes* (Ntougias et al., 2004; Zhang et al., 2014; Liang et al., 2015) were also present with relatively high abundance in the mushroom substrate, ensuring an effective simultaneous degradation system of lignocellulose in collaboration with the mushroom *G. lucidum*.

In our study, bacterial alpha diversity in the substrate increased significantly during the growth of *G. lucidum*. Bacterial alpha diversity indices (e.g., observed OTUs, Chao1, ACE and Shannon) peaked at the elongation stage. Vajna et al. (2010) attributed the higher diversity of bacterial community around the mushrooms to the growth of the mushrooms. Nevertheless, the evenness of bacterial community (Simpson index) remained unchanged with time. Bacterial community composition in the substrate changed significantly during the growth of *G. lucidum*. Particularly, bacterial community composition significantly differed between the elongation stage and the other three growth stages of *G. lucidum*, which might be linked to the changes in the substrate environment and the growth of *G. lucidum*.

LEfSe analysis revealed that a large number of bacterial taxa were enriched in the substrate at the elongation stage than at other growth stages of *G. lucidum*, including *Bacteroidetes* (e.g., genera *Flavobacterium* and *Sediminibacterium*), *Acidobacteria* (e.g., genus *Candidatus Koribacter*), *Nitrospirae* (e.g., genus *Nitrospira*), *Cyanobacteria* (e.g., order *Streptophyta*) and other taxa such as the orders of *Xanthomonadales* and *Rhodospirillales* and the family of *Comamonadaceae*. The increased relative abundance of these taxa were likely attributable to the colonization of these bacteria and the changes in the physicochemical condition of the substrate at the elongation stage of *G. lucidum*. These bacteria were characterized by their capacity in the biogeochemical cycling of major biogenic elements such as carbon, nitrogen, phosphorus and micronutrients. So the nitrogen, phosphorus and potassium and other nutrients in the substrate were likely more demanded by these bacteria at the elongation stage than at other stages. The reduction in substrate total nitrogen, phosphorus and potassium at the elongation stage highly supported this assumption. The activities of the bacterial community in the cultivation substrate significantly affect growth and quality of the edible mushrooms (Miller et al., 1990; Stölzer & Grabbe, 1991). Nitrogen fixation genes were detected in the genus of *Burkholderia* (Minerdi et al., 2001). *Nitrospirae* plays important roles in nitrification during the mushroom cropping process (Mcgee et al., 2017). Other bacteria may provide essential growth factors such as thiamine (Henningsson, 1967). The enrichment of these bacteria and the increased bacterial diversity at the elongation stage might be important to trigger the development of the fruiting bodies of *G. lucidum.*

*G. lucidum* produces various enzymes to degrade lignin and synthesize substances to accomplish its growth as other white rot fungi (Coelho et al., 2010). Accordingly, the changes in the physicochemical properties of the substrate materials by *G. lucidum* during its growth might be an important driver of the successional changes in bacterial community composition in the substrate. As the main ingredient of the substrate material, cottonseed hull can affect the growth of *G. lucidum* to a great extent. Previous studies reported compositional changes in a substrate with cottonseed hull, which included cellulose, hemicelluloses and lignin, during the growth period of mushrooms (Li, Pang & Zhang, 2001; Ni et al., 2002). The abundances of most bacterial groups were dependent on organic C and total N in the habitat (Zhang, 2010). In our study, the moisture content of substrate continued decreasing with the growth of *G. lucidum*, likely to be another key factor to affect the bacterial community composition and diversity. Therefore, the qualitative and quantitative change in the substrate physicochemical properties might have driven the dynamic shifts in the composition and functions of bacterial community during the growth of *G. lucidum*. In addition, bacterial communities can be directly affected by organic substances that fungi secrete (Frey-Klett, Pierrat & Garbaye, 1997). Metabolites of mushrooms such as carbohydrates, amino acids, thiols and all kinds of enzymes were secreted to change the physicochemical conditions in the substrate (Rangel-Castro, Danell & Pfeffer, 2002; Zhu et al., 2013), likely creating a suitable environment for bacterial colonization and growth at the later stage. In this study, differentially abundant bacterial taxa were uncovered in the substrate at different growth stages, most likely associated with the differed metabolic functions in the community.

The reconstruction of metagenome based on marker genes allows inference of the potential functional profile of the bacterial community in the substrate of *G. lucidum*. Of the identified KEGG pathways, 17.24% were related to environmental information processing, in which membrane transport dominated. Membrane transport of the bacterial community was predicted more abundant at the budding and hyphal stages than at the elongation stage in the substrate. The changes in the predicted functional pathways in the bacterial communities were likely attributable to the changes in the substrate physicochemical conditions. The pathway of membrane transport showed high abundance when the moisture content was high at the early stage. The extracellular enzymes and other organic substances that microbes produce are transported by liquid phase diffusion (Yang et al., 2011). High moisture content facilitates the transport of nutrients and mobility of bacteria, increasing the availability of nutrients to bacteria and intercommunication between microorganisms in the substrate. For bacteria, outer membrane proteins play essential roles in bacterial adaptation (Lin, Huang & Zhang, 2002). The membrane compositions differ from bacterial species (Sohlenkamp & Geiger, 2016). The outer membranes are important for bacterial adaption, as they are associated with the uptake of water, energy and nutrient, microbial interaction regulations and stress-resistance (Lin, Huang & Zhang, 2002; Schwechheimer & Kuehn, 2015). Outer-membrane vesicles make bacteria interact with the environment and survive under stress conditions (Schwechheimer & Kuehn, 2015). Therefore, in addition to the effect of *G*. *lucidum,* the high abundance of predicted environmental information processing pathway (particularly membrane transport) in the bacterial community at the early stage of *G*. *lucidum* was also likely related to the adaptation and colonization mechanisms of the bacterial settlers that entered into the substrate from air.

At the elongation stage, the abundance of the predicted membrane transport and xenobiotics biodegradation and metabolism pathways reduced whereas the predicted genetic information processing pathways (e.g., replication and repair, and translation), metabolism pathways (e.g., carbohydrate metabolism, energy metabolism, enzyme families, glycan biosynthesis and metabolism, and metabolism of cofactors and vitamins) increased, suggesting a functional change in the bacterial community from environmental adaptation and survival strategies to self-development with the growth of *G. lucidum*. The increase of predicted translation pathway at the elongation stage might be a result of rapid protein synthesis in bacterial cells in response to the rapid growth of *G*. *lucidum*. At this stage, bacterial richness and diversity increased, supported by the evidence of the reduction of the nutrients (e.g., total nitrogen, phosphorus and potassium) and moisture content in the substrate. Thus, the sufficient provision of nutrients and water to the substrate at the elongation stage might be important to sustain the growth and metabolic activities of the fungus and the substrate -associated microbiome.

Microbes are ubiquitous and participate in the life activities of other organisms (Singh, 2015). It was noteworthy that those bacterial OTUs that were present throughout the whole life cycle of *G. lucidum* accounted for more than 94% of the bacterial community, indicating that they were the first settlers and continued to be present in the substrate. These bacterial OTUs might have great capacity to cope with the acidity of the substrate environment and the resource competition with *G. lucidum* and other organisms. Some of these OTUs might also be beneficial to the growth of *G. lucidum*. Bacteria were involved in cell membrane permeability, metabolic activity, exudates absorption and nutrient bioavailability of mycelia, affecting the fruiting body growth (Fitter & Garbaye, 1994; Frey-Klett, Pierrat & Garbaye, 1997). For instance, bacterial metabolites were found related to fruiting body formation of *Agaricus bisporus* and help the mushroom resist pathogens (Urayama, 1967; Park & Agnihotri, 1969; Zhang, 1999). Bacteria such as fluorescent pseudomonads were particularly beneficial to *Pleurotus ostreatus* budding, which brings up a potential research direction for improving the yield and quality of *Ganoderma lucidum* by inoculating beneficial bacteria (Ouhdouch, Barakate & Finance, 2001; Cho et al., 2003). The phyla *Actinobacteria* and *Firmicutes* were suggested to carry out the conversion of wheat straw into utilisable sugars (Mcgee et al., 2017). In our study, a higher abundance of *Bacillus* was observed at the early growth stages (e.g., hyphal stage and budding stage) of *G. lucidum*. *Bacillus* spp. isolated from the cultivation substrate can optimize the growth of *Pleurotus ostreatus* by inhibiting *Trichoderma harzianum* growth through laccase induction (Velázquez-Cedeño et al., 2008). *Bacillus* provided relative protection mechanism against other harmful fungi because mycelia of cultivated mushroom were easily contaminated by other harmful fungi before primordia formation. Hence, the potential enhancement of bacterial activities on the growth of *G. lucidum* is worth further investigation.

## Conclusions

The study revealed a significant effect of *G. lucidum* growth on the physicochemical properties and bacterial community composition of the substrate materials (e.g., pH, moisture and nutrient conditions). Changes in the substrate physicochemical properties associate with the growth of *G. lucidum* were likely the direct factor that drove the changes in bacterial community composition. The diversity, structure and the predicted functional pathways of the substrate bacterial community greatly differed between the elongation stage and other growth stages. The decrease of nutrient contents and moisture indicated a rigorous growth of *G. lucidum* at the elongation stage. We therefore suggest that the nutrient supply especially at the elongation stage is crucial to the growth of *G. lucidum,* the diversity and the metabolic activities of the bacteria in the substrate. Hence, this study increased the understanding of the *G. lucidum*-bacteria-substrate interactions to hopefully facilitate the industrial cultivation of *G. lucidum.*

##  Supplemental Information

10.7717/peerj.4975/supp-1 Table S1 High quality read distribution between samples

10.7717/peerj.4975/supp-2 Table S2 The average relative abundance of different genera (relative abundance > 1% in at least one growth stage) in the substrate during all of the growth stages of *G. lucidum*

10.7717/peerj.4975/supp-3 Figure S1 The mixed substrate, composed of cottonseed hull (90%), wheat bran (5%), corn flour (4%) and gypsum (1%), before being put into polypropylene cultivation bags Photo by Xiaolin Li.

10.7717/peerj.4975/supp-4 Figure S2 Inoculation of *G. lucidum* in a laminar flow cabinet after sterilization Photo by Xiaolin Li.

10.7717/peerj.4975/supp-5 Figure S3 The cultivation site of *G. lucidum* Photo by Xiaolin Li.

10.7717/peerj.4975/supp-6 Figure S4 Sample collection in the clean bench Photo by Xiaolin Li.

10.7717/peerj.4975/supp-7 Figure S5 A diagram of metabolic functional prediction on bacterial community in the substrate of *G. lucidum* Different microbial metabolic functions were arranged vertically in accordance with their respective modules, in which the length of the graph revealed the abundance of bacterial genes related to the corresponding functions in the sample.

## Additional Information and Declarations

### Data Availability

The following information was supplied regarding data availability: NCBI: accession numbers are SRR5801759–SRR5801768 and SRR5801783, SRR5801784 in BioProject ID PRJNA392763.
