# Supplementary material for: Dynamic succession of substrate-associated bacterial composition and function during Ganoderma lucidum growth
Source: PeerJ. 2018 Jun 13;6:e4975. doi: 10.7717/peerj.4975 (PMC6004108; doi:10.7717/peerj.4975)
Supplement: Table S1 [file peerj-06-4975-s001.docx]

| Sample | Replicate 1 | Replicate 2 | Replicate 3 |
| --- | --- | --- | --- |
| Hyphal stage | 54,662 | 49,181 | 49,930 |
| Budding stage | 69,618 | 66,014 | 65,561 |
| Elongation stage | 43,333 | 35,896 | 34,095 |
| Mature stage | 46,829 | 25,996 | 49,583 |
