# Supplementary material for: Dynamic succession of substrate-associated bacterial composition and function during Ganoderma lucidum growth
Source: PeerJ. 2018 Jun 13;6:e4975. doi: 10.7717/peerj.4975 (PMC6004108; doi:10.7717/peerj.4975)
Supplement: Table S2 [file peerj-06-4975-s002.docx]

| Phylum | Genus | Abu. (%) at hyphal stage | Abu. (%) at budding stage | Abu. (%) at elongation stage | Abu. (%) at mature stage |
| --- | --- | --- | --- | --- | --- |
| Acidobacteria | An unclassified genus of Acidobacteria (p) | 2.91 | 1.99 | 5.20 | 0.02 |
| Actinobacteria | *Rhodococcus* | 0.47 | 0.26 | 1.87 | 1.63 |
| Bacteroidetes | *Sediminibacterium* | 1.59 | 0.96 | 8.31 | 1.15 |
| Cyanobacteria | An unclassified genus of *Streptophyta* (o) | 0.00 | 0.00 | 1.26 | 0.01 |
| Firmicutes | *Bacillus* | 12.95 | 11.31 | 1.85 | 0.31 |
|  | *Geobacillus* | 2.18 | 1.89 | 4.41 | 3.41 |
|  | *Enterococcus* | 9.58 | 8.88 | 1.31 | 0.04 |
|  | *Lactococcus* | 9.95 | 8.52 | 12.18 | 10.25 |
|  | *Streptococcus* | 0.77 | 0.65 | 1.52 | 1.33 |
|  | *Alkaliphilus* | 1.81 | 1.28 | 0.19 | 0.00 |
| Proteobacteria | An unclassified genus of *Caulobacteraceae* (f) | 2.78 | 7.34 | 2.51 | 1.32 |
|  | *Brevundimonas* | 0.05 | 0.07 | 0.24 | 2.87 |
|  | An unclassified genus of *Rhizobiales* (o) | 0.70 | 2.04 | 0.18 | 0.04 |
|  | An unclassified genus of *Bradyrhizobiaceae* (f) | 2.40 | 3.96 | 2.20 | 22.60 |
|  | *Ochrobactrum* | 0.86 | 0.66 | 1.14 | 0.57 |
|  | *Agrobacterium* | 29.77 | 20.57 | 0.10 | 0.26 |
|  | An unclassified genus of *Rhodospirillaceae* (f) | 0.30 | 0.85 | 1.62 | 0.04 |
|  | *Burkholderia* | 8.09 | 15.98 | 7.70 | 0.72 |
|  | An unclassified genus of *Comamonadaceae*(f) | 1.27 | 1.13 | 2.55 | 0.82 |
|  | *Ralstonia* | 1.30 | 1.60 | 5.79 | 4.73 |
|  | An unclassified genus of *Enterobacteriaceae*(f) | 1.71 | 1.43 | 4.41 | 19.24 |
|  | *Acinetobacter* | 0.25 | 0.17 | 1.08 | 8.49 |
|  | An unclassified genus of *Pseudomonadaceae*(f) | 0.18 | 0.42 | 0.36 | 3.53 |
|  | *Pseudomonas* | 0.76 | 0.69 | 1.97 | 6.85 |
|  | *Rhodanobacter* | 0.43 | 0.48 | 3.37 | 0.01 |
