# Supplementary figures and images for: Dynamic succession of substrate-associated bacterial composition and function during Ganoderma lucidum growth
Source: PeerJ. 2018 Jun 13;6:e4975. doi: 10.7717/peerj.4975 (PMC6004108; doi:10.7717/peerj.4975)

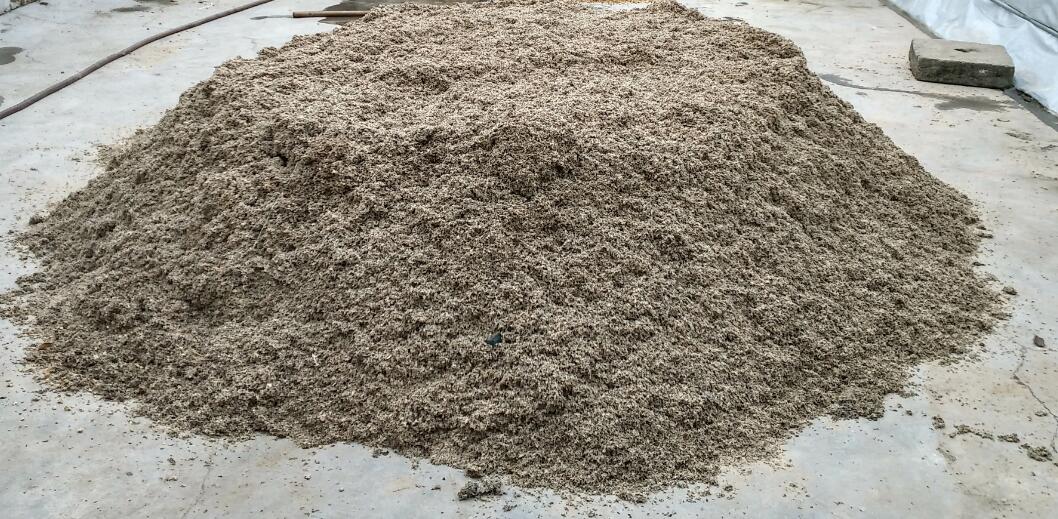

Supplement: Figure S1 — Photo by Xiaolin Li. [file peerj-06-4975-s003.jpg]

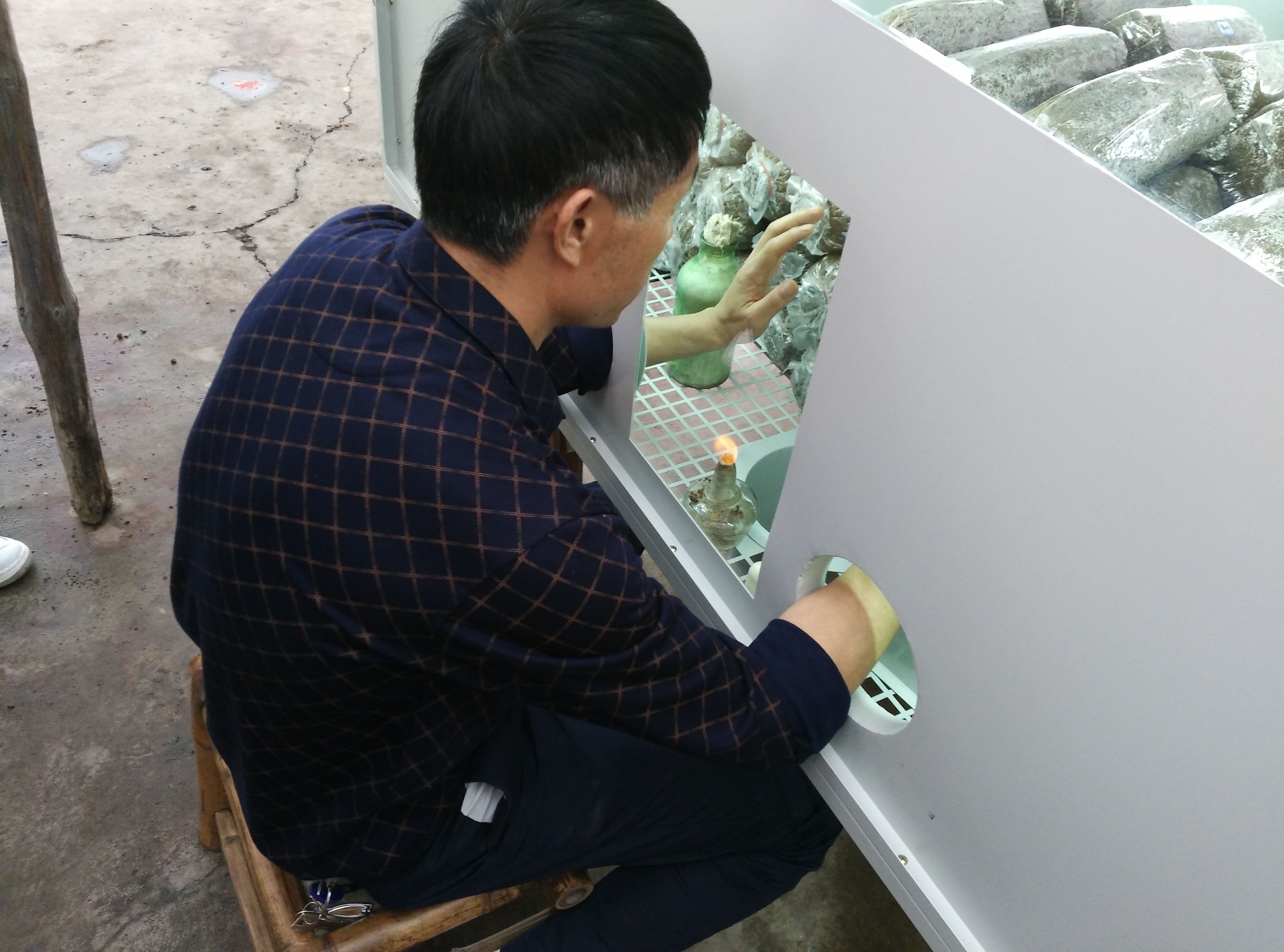

Supplement: Figure S2 — Photo by Xiaolin Li. [file peerj-06-4975-s004.jpg]

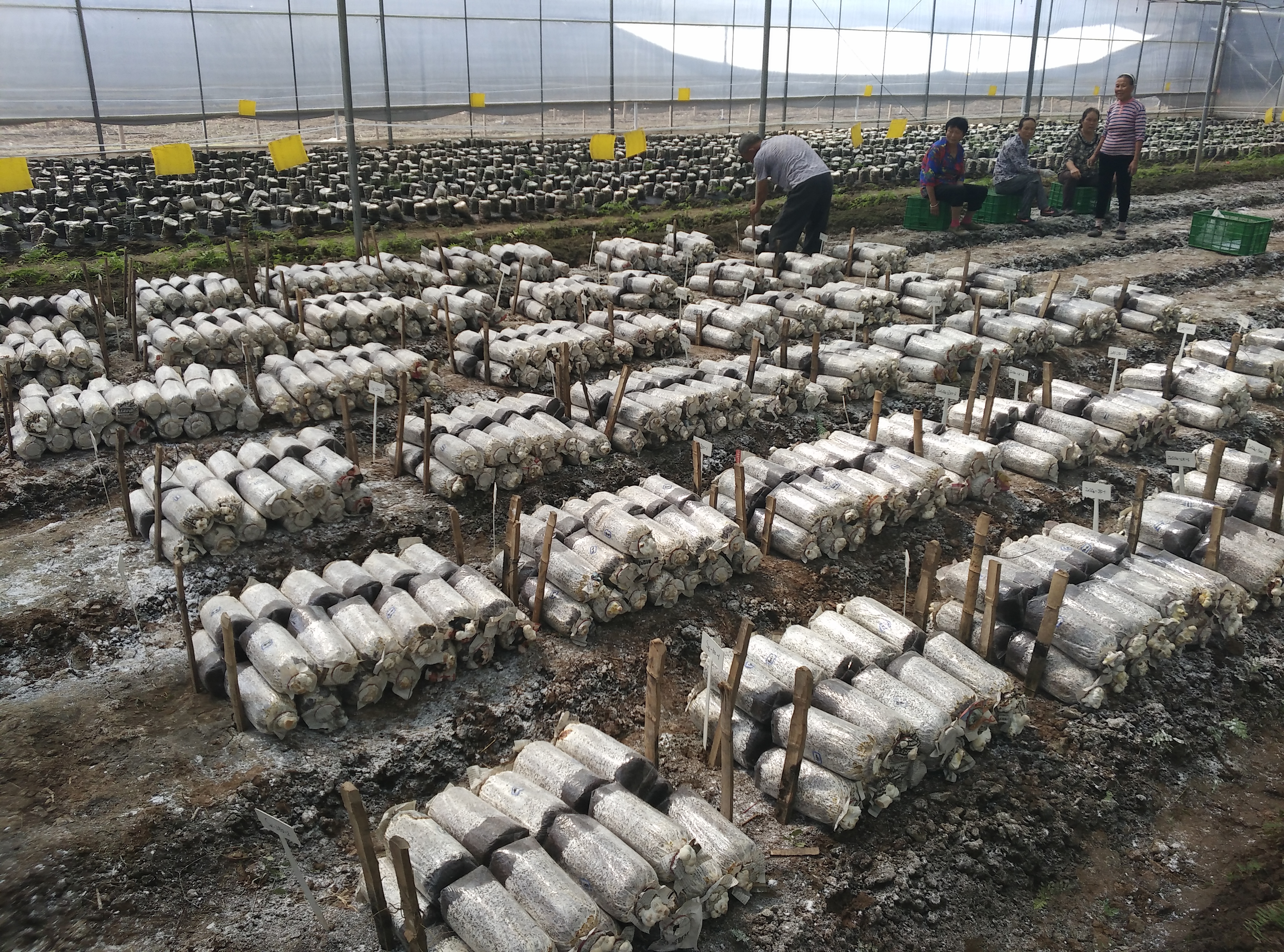

Supplement: Figure S3 — Photo by Xiaolin Li. [file peerj-06-4975-s005.jpg]

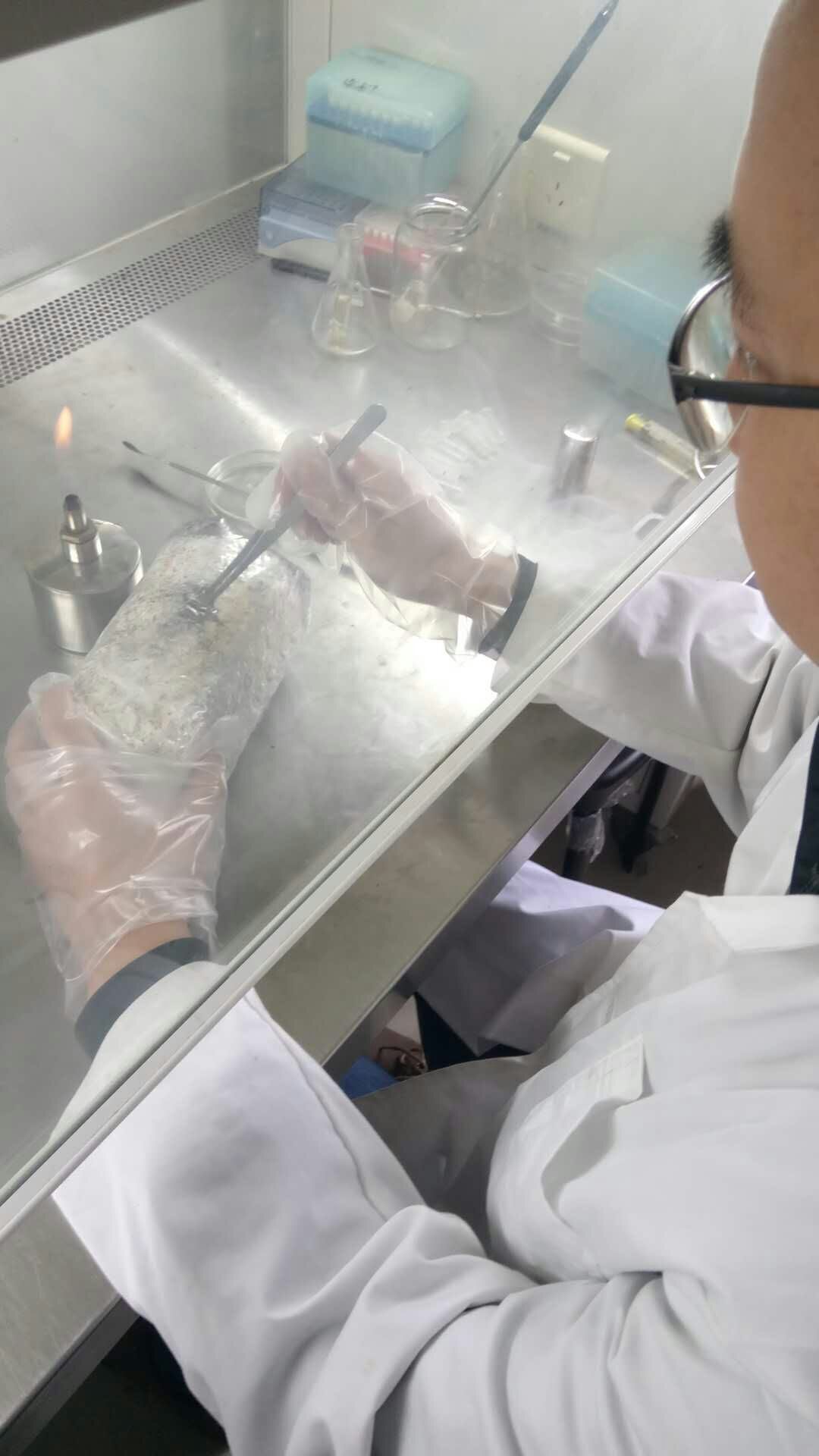

Supplement: Figure S4 — Photo by Xiaolin Li. [file peerj-06-4975-s006.jpg]

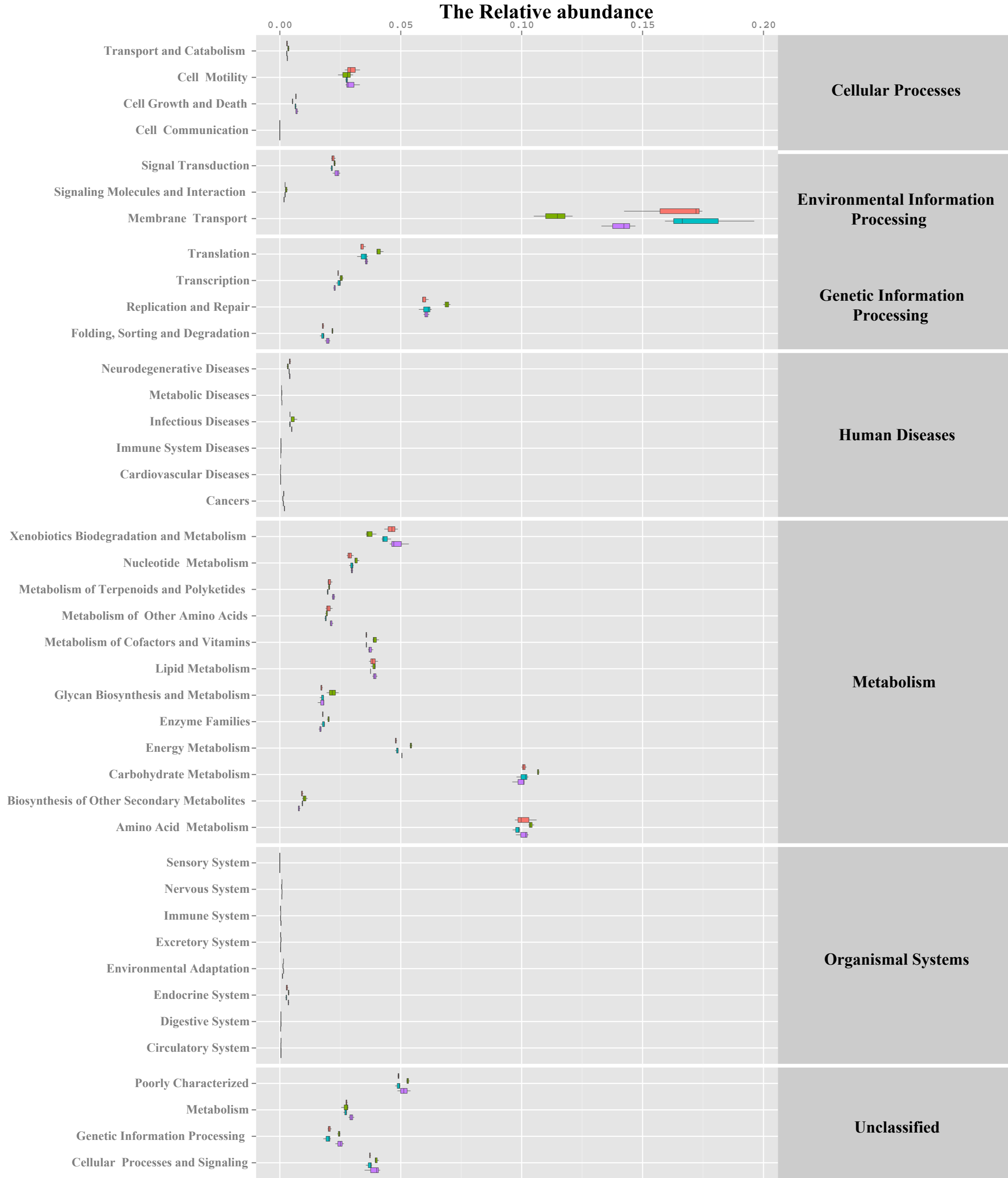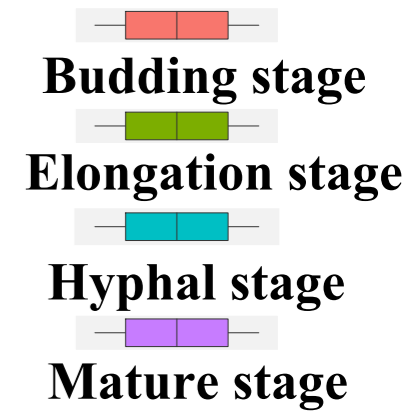

Supplement: Figure S5 — Different microbial metabolic functions were arranged vertically in accordance with their respective modules, in which the length of the graph revealed the abundance of bacterial genes related to the corresponding functions in the sample. [file peerj-06-4975-s007.pdf]
